# Influence of Preoperative Depression on Pain, Function, and Complications After Total Ankle Arthroplasty: A Systematic Review

**DOI:** 10.3390/jcm14197080

**Published:** 2025-10-07

**Authors:** Iosafat Pinto, Panagiotis Konstantinou, Lazaros Kostretzis, Tryfon Ditsios, Chrysanthos Chrysanthou, Anastasios P. Nikolaides, Stylianos Kapetanakis, Konstantinos Ditsios

**Affiliations:** 1 Orthopaedics and Trauma, General Hospital of Imathia, Health Unit of Veria, 59132 Veria, Greece; pintoiosif@outlook.com; 2 2nd Orthopaedic Department, Aristotle University of Thessaloniki, Eth. Aminis 41, 54635 Thessaloniki, Greece; 3 Military Service, Greek Army, 85111 Megisti, Greece; 4 Occupational Therapy Department, University of Western Macedonia, 50150 Kozani, Greece; 5 University Hospitals Birmingham NHS Foundation Trust, Birmingham B7 5TE, UK

**Keywords:** total ankle arthroplasty, depression, outcomes, complications, patient-reported outcomes, systematic review

## Abstract

**Background:** Depression has been identified as an important determinant of outcomes in hip and knee arthroplasty, but its impact on total ankle arthroplasty (TAA) remains unclear. Given the growing use of TAA as a treatment for end-stage ankle arthritis, understanding psychosocial risk factors is critical for optimizing surgical outcomes. This study aims to assess the effect of preoperative depression on clinical and functional outcomes following total ankle arthroplasty. **Methods:** A systematic review was conducted in accordance with PRISMA guidelines and prospectively registered with the Open Science Framework. PubMed, Cochrane Library, and CINAHL were searched through August 2025 for studies reporting outcomes of TAA stratified by depression status. Eligible designs included randomized trials, cohort studies and case series. Risk of bias was assessed using the Newcastle–Ottawa Scale (NOS). Given heterogeneity in study designs, depression definitions, and outcome measures, findings were synthesized narratively and summarized using a revised effect-direction plot. **Results:** Six unique studies involving approximately 9000 patients met inclusion criteria. Five studies were rated as good quality on the Newcastle–Ottawa Scale, while one study was judged to be of moderate quality. Four studies assessing pain outcomes consistently demonstrated worse postoperative pain or less improvement in patients with depression. Three of five studies assessing functional or disability outcomes reported reduced improvement, while two studies found no independent association. Two studies evaluating complications showed higher risks of adverse events, including prolonged hospital stay, non-home discharge, osteophytosis, and implant subsidence, among depressed patients. Revised effect-direction synthesis confirmed a consistent trend toward poorer outcomes across pain, function, and complication domains. **Conclusions:** Depression is associated with worse pain and higher complication rates following TAA, while its influence on functional recovery was not demonstrated uniformly. These findings support the importance of routine preoperative screening and targeted management of depression. Further prospective, multicenter studies and interventional trials are needed to clarify causality and optimize perioperative care.

## 1. Introduction

Total ankle arthroplasty (TAA) has emerged as an increasingly utilized surgical option for end-stage ankle arthritis, offering the potential to restore pain-free mobility and preserve ankle kinematics compared with arthrodesis [1]. Over the past two decades, advances in implant design, surgical technique, and patient selection have contributed to improved survival rates and functional outcomes [2]. Nonetheless, outcomes following TAA remain more variable compared with hip or knee arthroplasty, and patient satisfaction rates are not universally high [3]. This has prompted growing interest in understanding patient-specific factors that may influence prognosis.

Among such factors, psychological comorbidities—particularly depression—have received increasing attention in orthopedic research. Depression is prevalent in patients with chronic musculoskeletal conditions, with estimates ranging from 15% to 30% depending on population and screening instrument [4]. Comparable rates have been observed in hip and knee arthroplasty cohorts [5,6], where depression has also been linked to persistent pain, slower functional recovery, increased opioid requirements, higher complication rates, and reduced satisfaction. In total ankle arthroplasty, Wilson et al. reported a prevalence of approximately 11% [7]. Similarly, in shoulder arthroplasty, Lunati et al. found that 14% of patients had preoperative depression, which was associated with increased complication rates and healthcare utilization [8]. Collectively, these data underscore that depression is a common comorbidity across endoprosthetic procedures. However, in contrast to the extensive evidence in hip, knee, and shoulder arthroplasty, the role of depression in TAA remains underexplored, despite the parallels in perioperative rehabilitation demands.

Its impact has been well documented in large joint arthroplasty, where patients with depression undergoing total knee or hip replacement are more likely to experience persistent pain, slower functional recovery, increased opioid requirements, higher complication rates, and reduced satisfaction [5,6]. Yet, despite the parallels in perioperative rehabilitation demands, the influence of depression on TAA outcomes has been far less extensively investigated.

The objective of this systematic review was to evaluate the impact of preoperative depression on outcomes following total ankle arthroplasty. We examined whether patients with depression experience differences in postoperative pain, functional recovery, and complication rates compared with their non-depressed counterparts. By systematically reviewing and synthesizing the available evidence, we aimed to provide a comprehensive assessment of the role of depression in TAA outcomes and to highlight areas where further research is most urgently needed.

## 2. Methods

This systematic review was conducted in accordance with the Preferred Reporting Items for Systematic Reviews and Meta-Analyses (PRISMA) guidelines. The protocol was prospectively registered with the Open Science Framework (OSF; DOI: 10.17605/OSF.IO/S2NCJ (accessed on 15 September 2025)) prior to data collection and analysis. The review question was framed using the Population, Intervention, Comparison, Outcome (PICO) approach. The population of interest consisted of adult patients undergoing total ankle arthroplasty. The exposure was the presence of preoperative depression, either as a formal diagnosis recorded in medical records or administrative coding, or as depressive symptoms identified through validated screening tools. The comparator group comprised patients without depression. The outcomes of interest included patient-reported measures of pain, function or disability, and health-related quality of life, as well as adverse events such as postoperative complications, readmission, discharge disposition, and length of hospital stay.

A systematic search of PubMed, Cochrane Library, and CINAHL was performed from database inception through August 2025. The following Boolean query was used: (arthroplasty OR replacement) AND ankle AND (outcome OR complication) AND depression. This search strategy was adapted for each database. The search was restricted to studies published in English.

Eligible studies were defined according to the following criteria: (1) randomized controlled trials, prospective or retrospective cohort studies, or case series; (2) adult patients (≥18 years) undergoing primary total ankle arthroplasty for end-stage ankle arthritis; (3) depression assessed either through a documented clinical diagnosis (medical records or administrative coding) or validated screening tools; and (4) outcomes reported stratified by depression status in at least one relevant domain, including pain, function/disability, health-related quality of life, or complications such as readmission, discharge disposition, or length of stay. Studies were excluded if they (1) did not stratify results according to depression status; (2) evaluated ankle arthrodesis only; (3) were case reports, narrative or systematic reviews, or conference abstracts; (4) were non-peer-reviewed publications; (5) were not available in full text; (6) were not published in English; or (7) were animal or cadaveric studies.

Titles and abstracts were screened independently by two reviewers (I.P and P.K), followed by full-text review of potentially eligible studies. To maximize comprehensiveness, reference lists of included studies and relevant reviews were manually screened for additional eligible articles. Disagreements were resolved through discussion until consensus was reached.

Data extraction was conducted independently by two reviewers (I.P and P.K) using a predesigned standardized template. Information collected included study characteristics (author, year, country, design, sample size, and follow-up period), patient demographics (mean age, sex distribution), the method used to define or assess depression, the type of outcome measures employed, and study findings related to depression. Interrater reliability for study selection was assessed using Cohen’s kappa [9]. Values above 0.80 were interpreted as almost perfect agreement, 0.61–0.80 as substantial, 0.41–0.60 as moderate, 0.21–0.40 as fair, and 0.20 or lower as slight agreement.

Risk of bias for included studies was assessed using the Newcastle–Ottawa Scale (NOS), which evaluates three domains: selection, comparability, and outcome assessment [10] Each study was independently assessed by two reviewers (I.P and L.K) and categorized as having low (≥7 stars), moderate (5–6 stars), or high risk of bias (<5 stars).

Given the heterogeneity of study designs, definitions of depression, and outcome measures, a quantitative meta-analysis was not methodologically appropriate. Instead, findings were synthesized narratively and summarized visually using a revised effect-direction plot [11]. This approach allows evidence to be aggregated when statistical pooling is not feasible, by displaying whether preoperative depression was associated with worse, better, or no difference in outcomes across studies. A single revised effect-direction plot was generated, incorporating three outcome domains—pain, functional outcomes, and complications—to provide a structured overview of the consistency of associations in the available literature. No statistical conversions were undertaken, and data were reported as presented in the original studies. Missing summary statistics were not imputed. Outcomes from individual studies were organized by domain and summarized in tables detailing study characteristics, and principal findings.

## 3. Results

### 3.1. Study Selection

**Figure 1:**
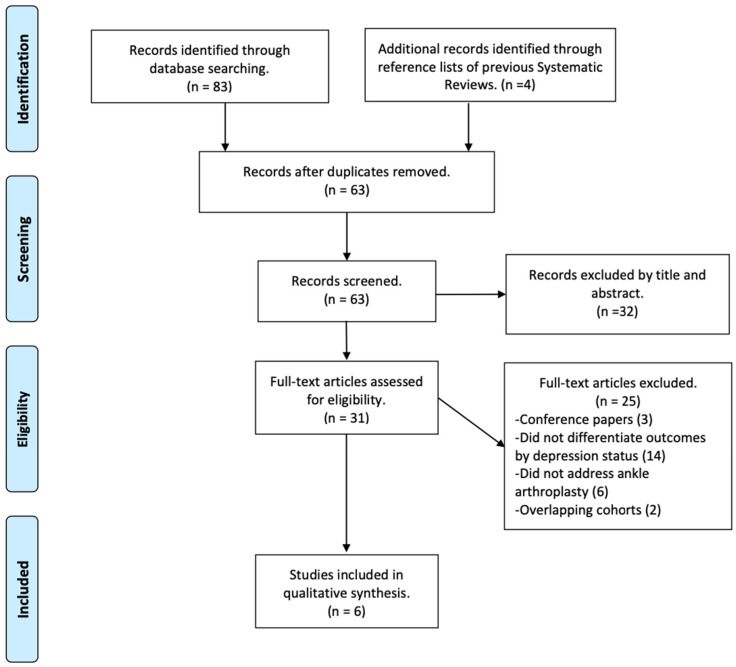
PRISMA flow diagram.

The database search yielded a total of 83 records. After removal of duplicates, 63 unique titles and abstracts were screened for eligibility. Of these, 31 articles were selected for full-text review. Following detailed assessment, six studies met the predefined eligibility criteria and were included in the final synthesis. The study selection process is summarized in the PRISMA flow diagram (Figure 1). Agreement between reviewers was high across screening stages, with a kappa of 0.823 during title and abstract screening and 0.842 during full-text review, both indicating almost perfect agreement.

### 3.2. Characteristics of Included Studies

The six included studies [7,12,13,14,15,16] were published between 2020 and 2025 and together involved approximately 9000 patients undergoing primary total ankle arthroplasty. Study designs included multicenter registry-based cohorts, single-institution retrospective series, and prospective observational cohorts. Follow-up periods ranged from one year to five years. Depression was identified either through diagnostic codes and medical record review or through validated screening instruments. The operational definition of depression therefore varied, with some studies relying on clinical diagnosis and others on administrative codes. A detailed summary of study characteristics is provided in Table 1.

### 3.3. Risk of Bias

Risk of bias assessment using the Newcastle–Ottawa Scale indicated that the majority of included studies were of good methodological quality (Table 2). Five of the six unique studies scored seven or more stars, reflecting adequate definition of patient cohorts, use of validated outcome measures, and generally complete follow-up. One study [7], based on administrative data, scored lower (six stars) due to limited adjustment for confounding variables and reliance on diagnostic coding. The most consistent methodological limitations across studies were the predominance of retrospective designs, the absence of multivariable adjustment for relevant comorbidities in all but one registry-based analysis [13], and heterogeneity in the definition and measurement of depression. Despite these issues, the overall evidence base can be considered moderate-to-good quality, lending reasonable confidence to the observed associations.

### 3.4. Pain Outcomes

Four studies reported outcomes related to postoperative pain in patients with and without preoperative depression (Table 3). Across these, a consistent trend was observed in which depression was associated with higher pain scores or less pain improvement following total ankle arthroplasty; nevertheless, in all studies patients with depression still experienced significant reductions in pain from preoperative to postoperative assessments. Cunningham et al. [16], demonstrated that lower preoperative Short Form-36 Mental Component Summary (SF-36 MCS) scores, reflecting worse mental health, were predictive of higher postoperative pain scores. Kim et al. [15] similarly found that depressed patients reported significantly worse pain relief following surgery. In addition, Yasui et al. [12] reported that patients with lower preoperative depression score had less pain at 6 months and 1 year postoperatively. Wang et al. [14] also observed that patients at high risk of depression had significantly higher pain scores at final follow-up compared with those at low risk. Collectively, these findings suggest a consistent association between depression and suboptimal pain outcomes following TAA, with only minor variation in the strength of the reported associations.

### 3.5. Functional and Disability Outcomes

Five studies provided data on functional or disability outcomes (Table 4). Cunningham et al. [16] reported that lower preoperative SF-36 MCS scores were predictive of poorer improvement in functional outcomes as measured by the Short Musculoskeletal Function Assessment (SMFA) and Short Form-36 Physical Component Summary (SF-36 PCS). Kim et al. [15] reported that patients with depressive symptoms had significantly lower postoperative American Orthopaedic Foot & Ankle Society score (AOFAS) compared with non-depressed patients. Similarly, Wang et al. [14] found that at the final follow-up depression was associated with less improvement in functional scores. It is worth noting that in all three studies where depressed patients had significantly lower functional scores compared with non-depressed patients, they nonetheless demonstrated significant improvement relative to their preoperative baseline.

Conversely, Wong et al. [13], in a registry analysis with adjustment for baseline physical and mental health, found no independent association between depression and postoperative function. Also, Yasui et al. [12] suggested that depressive tendencies do not affect postoperative functional results using objective assessment measures such as the Japanese Society for Surgery of the Foot score (JSSF) and the Timed Up and Go test (TUG). Overall, three out of five studies indicated worse functional outcomes in depressed patients, while the other two studies reported no independent association, highlighting some variability across study designs and adjustment strategies.

### 3.6. Complications and Adverse Events

Two studies assessed postoperative complication outcomes according to depression status. Wilson et al. [7] analyzing a large national database, found that preoperative depression was associated with significantly higher risks of non-home discharge, medical complications, prosthetic complication, wound complications, prosthetic joint infection and prolonged hospital stay. These associations remained consistent even after accounting for baseline demographics. Wang et al. [14] reported significantly higher complication rates among depressed patients undergoing total ankle arthroplasty, including osteophytosis and implant subsidence, although adjustment for confounding variables was not performed.

### 3.7. Effect-Direction Synthesis

**Figure 2:**
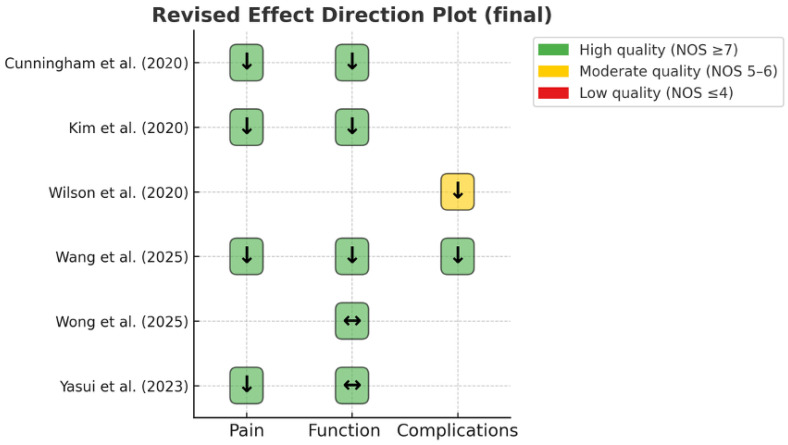
Revised Effect Direction Plot. Arrows: ↓ worse outcomes in depressed patients; ↔ no significant difference. Cells shaded by study quality using the Newcastle–Ottawa Scale (NOS): green = high (≥7), yellow = moderate (5–6), red = low (≤4) [7,12,13,14,15,16].

The revised effect-direction plot (Figure 2) demonstrated that four of the four studies assessing pain outcomes indicated worse results in depressed patients. Three of five studies assessing functional or disability outcomes suggested worse results, with two reporting no significant difference. Both studies reporting on complications demonstrated higher rates among depressed patients. When viewed collectively, the effect-direction synthesis indicates a consistent trend: depression is associated with worse pain, a greater likelihood of reduced functional improvement, and increased complications following total ankle arthroplasty.

## 4. Discussion

This systematic review synthesized evidence from six unique studies examining the influence of preoperative depression on outcomes after total ankle arthroplasty. Although the number of eligible studies was limited, the overall direction of evidence was consistent across different study designs and outcome measures. The revised effect-direction plot provided a clear overview, highlighting that depression was generally linked to less favorable results after surgery. Notably, in all studies where patient-reported outcomes were lower among depressed patients, these individuals still showed significant improvement compared with their own preoperative scores. This indicates that while depression may limit the extent of recovery, it does not preclude meaningful benefit from TAA and therefore should be regarded as a factor requiring recognition and support rather than a contraindication to surgery.

Beyond depression, several studies have highlighted the impact of other psychiatric disorders—such as anxiety, bipolar disorder, schizophrenia, and dementia—on outcomes after joint arthroplasty. These conditions have been associated with higher complication rates, longer hospital stays, and poorer functional recovery in hip, knee, and shoulder replacement cohorts [17,18,19]. Although our review focused specifically on depression in TAA, the broader mental health burden across endoprostheses underscores the importance of comprehensive psychosocial assessment.

### 4.1. Biological and Psychosocial Mechanisms

Several mechanisms could underlie the association between depression and poorer ankle arthroplasty outcomes. Biologically, depression has been linked to alterations in inflammatory pathways, hypothalamic–pituitary–adrenal axis dysfunction, and increased pain sensitivity, all of which may amplify the perception of postoperative pain and impair tissue healing [20,21,22]. Psychologically, depression is associated with reduced motivation, hopelessness, and catastrophizing, which may impair adherence to rehabilitation regimens and limit physical activity [23]. Socially, depression often co-occurs with socioeconomic disadvantage, unemployment, or social isolation, all of which can complicate recovery and access to postoperative care [24]. These multifactorial influences likely interact, producing a cumulative adverse impact on recovery trajectories after TAA.

### 4.2. Clinical Implications

The findings of this review carry several important implications for clinical practice. First, they underscore the need for systematic screening of depression in patients undergoing evaluation for total ankle arthroplasty. Validated tools such as the Patient Health Questionnaire-9 (PHQ-9) or the Hospital Anxiety and Depression Scale (HADS) can be easily administered in the preoperative setting and may help identify patients at risk. Second, integrating psychosocial optimization into perioperative care pathways may improve outcomes. Evidence from other surgical settings suggests that collaborative care models, cognitive behavioral therapy, and optimized pharmacologic management of depression can enhance postoperative recovery [25,26]. While no interventional trials have yet been conducted specifically in the TAA population, the consistency of associations observed in this review suggests that similar strategies may be beneficial. Third, counseling patients regarding the potential influence of depression on outcomes may improve shared decision-making and set realistic expectations for recovery.

### 4.3. Strengths and Limitations of the Review

Our review has several limitations, largely reflecting the characteristics of the included studies. These include the modest number of eligible publications, heterogeneity in the definitions of depression and outcome measures, and the predominance of retrospective observational designs. The reliance on aggregate published data also prevented patient-level analyses, which would have been valuable in clarifying interactions between depression and other prognostic factors. In addition, publication bias cannot be excluded, although the small number of studies precluded formal assessment. A further limitation is that several depression-related factors were not systematically addressed in the included studies. Other psychiatric comorbidities such as anxiety or bipolar disorder may also influence recovery trajectories, yet these were not consistently evaluated. In addition, depression is often associated with poorer nutritional habits and with the use of antidepressant medications, both of which could affect the postoperative outcomes. Since none of the available studies accounted for these variables, residual confounding cannot be excluded.

Despite these limitations, the review has notable strengths. It was conducted in accordance with PRISMA guidelines, prospectively registered in OSF, and based on a comprehensive multi-database search strategy. The use of the revised effect-direction plot enabled a structured synthesis of heterogeneous findings, while the inclusion of relatively recent studies—generally of good methodological quality—ensured relevance to contemporary surgical practice and implant design. Finally, an important strength is that, to our knowledge, this is the first systematic review to specifically evaluate the influence of depression on outcomes following total ankle arthroplasty.

### 4.4. Future Directions

Future research should focus on prospective, adequately powered cohort studies that incorporate standardized assessments of depression and adjust for key confounding variables. Ideally, multi-institutional or registry-based efforts would provide more generalizable findings. Interventional studies are also warranted to determine whether preoperative screening and targeted treatment of depression can improve outcomes after TAA. Finally, qualitative research exploring patient perspectives on how depression influences recovery may provide additional insights for tailoring perioperative support.

## 5. Conclusions

This systematic review provides the first synthesis of available evidence on the role of depression in outcomes following total ankle arthroplasty. Across six unique studies, depression was consistently associated with worse postoperative pain, reduced functional recovery in several but not all analyses, and higher complication rates. Although the overall evidence base remains modest and methodologically heterogeneous, the convergence of findings suggests that depression is an important risk factor for less favorable recovery trajectories after TAA. Importantly, however, patients with depression still demonstrate meaningful improvement compared with their preoperative status, indicating that depression is not a contraindication to TAA but a factor warranting recognition, counseling, and support. These results highlight the need for routine psychosocial screening, incorporation of mental health support into perioperative care, and further high-quality prospective studies to clarify causal mechanisms and evaluate targeted interventions.

## Figures and Tables

**Table 1:** Characteristics of included studies.

Study	Country	Level of Evidence	Study Design	Sample Size (n)	Sex (Females)	Depressed Patients (n)	Depression Assessment	Mean Follow-Up
Cunningham et al. (2020) [16]	USA	III	Retrospective cohort	612	302	129	ICD codes	1 to 2 years
Kim et al. (2020) [15]	South Korea	III	Retrospective comparative	40	18	13	CES-D, PHQ-9	24.3 ± 7.3 months
Wilson et al. (2020) [7]	USA	III	National database retrospective cohort	8049	3719	918	ICD codes	90 days
Yasui et al. (2023) [12]	Japan	IV	Retrospective cohort	20	19	5	HADS	1 year
Wong et al. (2025) [13]	UK	III	Retrospective cohort	98	38	35	EQ-5D-3L	2.1 ± 2.0 years
Wang et al. (2025) [14]	China	III	Retrospective comparative	66	46	25	HADS	34.35 ± 22.04 months

Values are presented as reported in the original studies. Continuous variables are expressed as mean ± standard deviation. Abbreviations: CES-D, Center for Epidemiologic Studies Depression Scale; PHQ-9, Patient Health Questionnaire-9; ICD, International Classification of Diseases; HADS, Hospital Anxiety and Depression Scale; EQ-5D-3L, EuroQol five-dimension three-level questionnaire.

**Table 2:** Newcastle–Ottawa Scale (NOS) assessment of included studies.

Study	Selection (Max 4)	Comparability (Max 2)	Outcome (Max 3)	Total (Max 9)
Cunningham et al. (2020) [16]	3	1	3	7
Kim et al. (2020) [15]	4	1	3	8
Wilson et al. (2020) [7]	3	1	2	6
Yasui et al. (2023) [12]	4	0	3	7
Wong et al. (2025) [13]	4	2	2	8
Wang et al. (2025) [14]	4	1	3	8

**Table 3:** Pain outcomes in patients with and without depression undergoing TAA.

Study	Measure of Pain	Preoperative		Statistical Significance of Difference (*p* Value) ^1^	Postoperative		Main Finding	Statistical Significance of Difference (*p* Value) ^2^
		Non-Depressed	Depressed		Non-Depressed	Depressed		
Cunningham et al. (2021) [16]	VAS	n/a	n/a	n/a	4 (1, 14)	9 (2, 28)	Depressed patients had higher postoperative pain and less improvement	<0.05
Kim et al. (2020) [15]	VAS	7.0 ±1.2	6.7 ± 2.2	0.154	1.4 ± 2.3	3.1 ± 2.4	Depressed group showed significantly less pain relief at 12 months	<0.001
Yasui et al. (2023) [12]	SAFE-Q pain subscale	42 (28–61)	32 (17–45)	n.s.	90 (82–97)	59 (40–87)	Higher depression scores linked to persistent postoperative pain	0.022
Wang et al. (2025) [14]	VAS	6.61 ± 0.39	6.85 ± 0.44	0.131	1.41 ± 0.48	3.42 ± 1.75	Depressed patients had higher postoperative pain	<0.001

Values of outcomes are reported as mean ± standard deviation, median (Q1, Q3), or median [IQR], according to the original study. Abbreviations: VAS, Visual Analog Scale; SF-36, 36-Item Short Form Health Survey; SAFE-Q, Self-Administered Foot Evaluation Questionnaire; n/a, non-applicable; n.s., non-significant. ^1^ *p*-value indicates difference between non-depressed and depressed patients preoperatively; ^2^ *p*-value indicates difference between non-depressed and depressed patients post-operatively.

**Table 4:** Functional outcomes in patients with and without depression undergoing TAA.

Study	Functional Measure	Preoperative		Statistical Significance of Difference (*p* Value) ^1^	Postoperative		Main Finding	Statistical Significance of Difference (*p* Value) ^2^
		Non Depressed	Depressed		Non Depressed	Depressed		
Cunningham et al. (2020) [16]	SMFA SF-36 PCS	n/a n/a	n/a n/a	n/a n/a	9.6 (3.7, 19.1) 75.2 (55, 86.8)	19.9 (8.1, 31.6) 51.2 (38.1, 73.6)	Depressed patients showed less functional improvement	<0.001
Kim et al. (2020) [15]	AOFAS	61.2 ± 14.2	54.4 ± 14.2	0.074	95.0 ± 8.1	89.3 ± 13.4	Depressed group had significantly poorer functional recovery	<0.001
Wong et al. (2025) [13]	MOXFQ	71.8±12.7	83.8 ± 12.9	0.008	25.7 ± 24.6	40.8 ± 30.1	After adjustment, no independent association between depression and function	0.42
Yasui et al. (2023) [12]	JSSF, SAFE-Q function, TUG	57 (54–61) NR 9.3 (7.2–12)	55 (54–58.25) NR 12 (11–13)	n.s. NR n.s.	NR	NR	Function domains unaffected by depression	>0.05
Wang et al. (2025) [14]	AOFAS	36.00 (27.00, 46.00)	38.67 ± 9.57	0.482	85.94 ± 6.54	75.00 ± 7.50	Worse functional outcomes in depressed patients	0.02

Values of outcomes are reported as mean ± standard deviation or median (Q1, Q3), according to the original study. Abbreviations: SMFA, Short Musculoskeletal Function Assessment; AOFAS, American Orthopaedic Foot & Ankle Society score; MOXFQ, Manchester–Oxford Foot Questionnaire; JSSF, Japanese Society for Surgery of the Foot score; SAFE-Q, Self-Administered Foot Evaluation Questionnaire; TUG, Timed Up and Go test; NR, values not numerically reported, only shown graphically.^1^ *p*-value indicates difference between non-depressed and depressed patients preoperatively; ^2^ *p*-value indicates difference between non-depressed and depressed patients post-operatively.

## Data Availability

No new data were created or analyzed in this study.
